# Exploring the Rare Case of a Sarcomatoid Variant of Urothelial Carcinoma

**DOI:** 10.7759/cureus.56679

**Published:** 2024-03-22

**Authors:** Sumithra A, Vallal Kani, Vimal Chander R

**Affiliations:** 1 Department of Pathology, Saveetha Medical College and Hospitals, Saveetha Institute of Medical and Technical Sciences, Saveetha University, Chennai, IND

**Keywords:** sarcomatoid variant, bladder cancer, immunohistochemistry, prognosis, urothelial carcinoma

## Abstract

A sarcomatoid variant of urothelial carcinoma (SVUC) is an extremely rare variant, which accounts for only 0.1-0.3% of all urothelial carcinomas of the bladder. SVUC is distinguished by the presence of biphasic components; there can be morphological and/or immunohistochemical substantiation of epithelial and mesenchymal differentiation. The patients with this variant have been associated with very poor disease-specific and overall survival rates in comparison with the high-grade pure urothelial carcinoma. Being a rare entity, it usually presents at a higher grade and is related to a dismal prognosis in comparison with conventional urothelial carcinoma. Careful examination, early diagnosis, and effective treatment are the most important steps for good survival. Here, we report a 58-year-old male who presented with complaints of hematuria for one and a half months with histopathology showing features of SVUC.

## Introduction

A sarcomatoid variant is an uncommon form of urothelial carcinoma, comprising less than 0.5% of all urothelial cancers in the bladder. Patients usually present with complaints of lower urinary tract symptoms and hematuria [1]. The tumor cells of the sarcomatoid variant have cytological, histological, and molecular properties of the biphasic components, epithelial (carcinoma) and mesenchymal (sarcoma), with the presence or absence of heterologous elements [2]. This rare biphasic variant of urothelial carcinoma has been associated with the worst prognosis and is considered a diagnostic challenge despite the utilization of ancillary tests [3]. The epithelial elements that react with uroplakin antibodies have been reported to show immunoreactivity in urothelial carcinoma; however, uroplakin II has more utility. The sarcomatoid variant of urothelial carcinoma (SVUC) does not carry any definitive treatment protocol, but the prognosis of the disease can be enhanced by radical surgical ablation and chemoradiation [2]. Here, we report an aggressive variant of urothelial carcinoma diagnosed by histopathological examination of a radical cystoprostatectomy specimen received in our hospital.

## Case presentation

**Figure 1:**
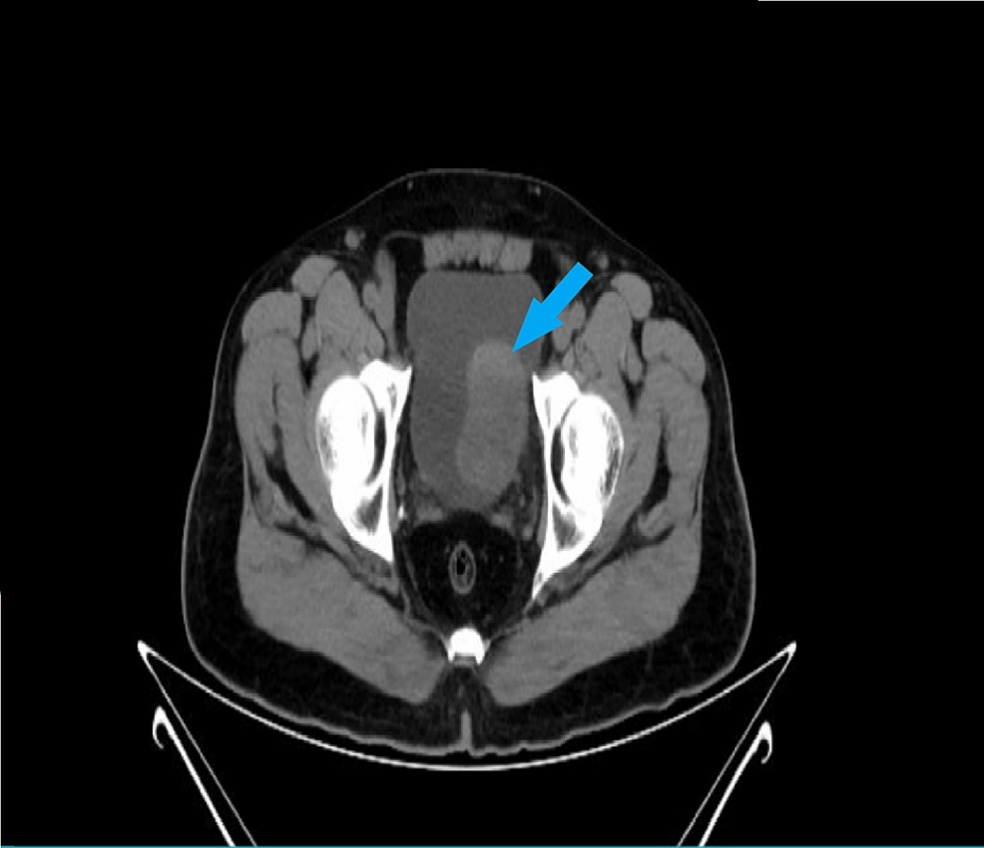
CT urography showing primary malignant neoplastic lesion of the urinary bladder. A large lobulated endophytic irregularly outlined heterogenous lesion of predominantly soft tissue attenuation protruding into the bladder lumen (arrow).

A 58-year-old male, with a known case of hypothyroidism, presented with complaints of hematuria for one and a half months, which was a full-stream, frank blood with amorphous sediment, associated with pain on and off. Also, he had lower abdominal pain for the past 10 days. There were no complaints of fever, nausea, vomiting, loss of appetite, loss of weight, loose stools, constipation, hematemesis, and melena. Vital signs were normal. The abdominal examination revealed tenderness over the lower abdominal region. All baseline investigations were done, which showed anemia with hemoglobin of 5.7 g/dl; hence, the patient was transfused with three units of blood to improve the hemoglobin level. An ultrasonogram showed a mass in the bladder, for which transurethral resection of bladder tumor (TURBT) was done. Grossly, multiple grey-white soft tissue fragments measuring 1.5×1×0.5 cm in the aggregate and histopathological examination revealed a high-grade sarcomatoid variant of invasive urothelial carcinoma with lamina propria invasion. CT urography was done, which revealed a primary malignant neoplastic lesion of the urinary bladder (Figure 1).

**Figure 2:**
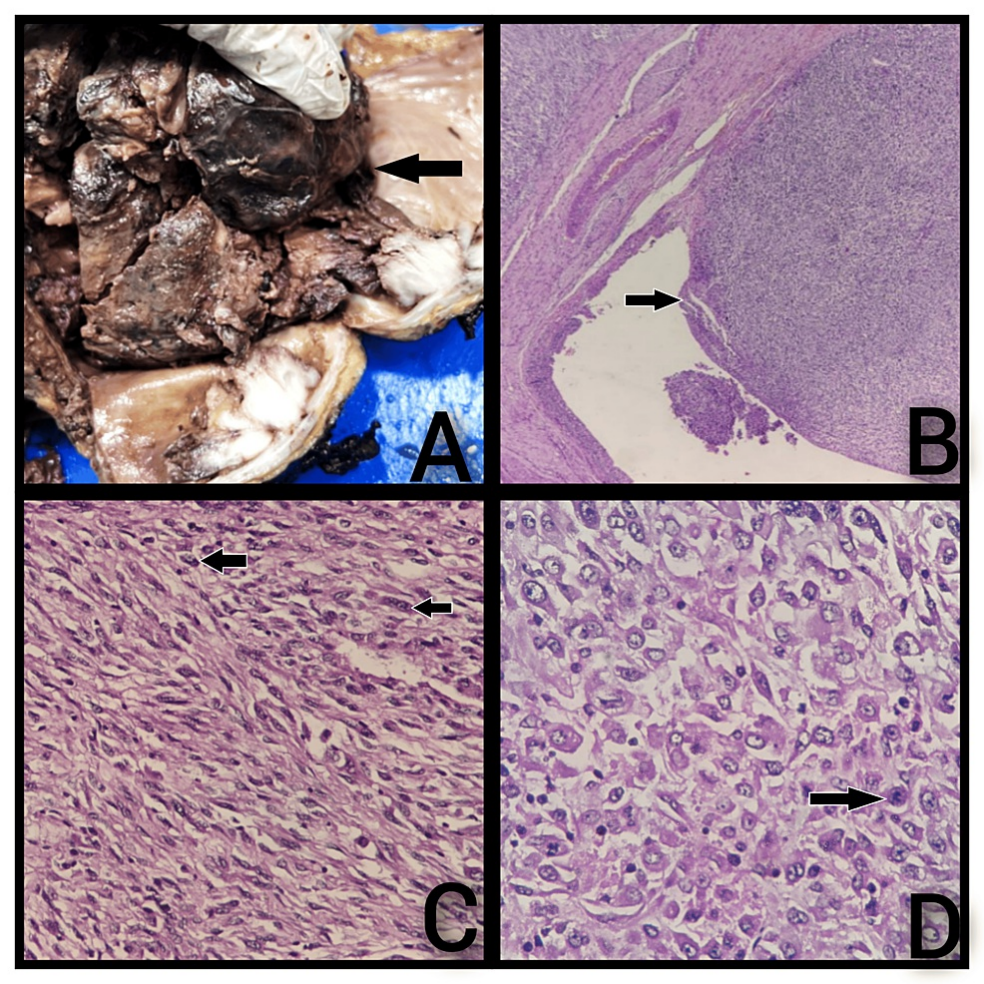
Gross and microscopy of sarcomatoid variant of urothelial carcinoma of the urinary bladder showing sheets of malignant cells with pleomorphic nuclei and mitoses. A: Gross - Large polypoid intraluminal growth measuring 12×11×9 cm arising from the right lateral wall of the bladder (arrow). B: Bladder wall lined by the urothelium with invasive malignant neoplasm showing sheets of spindle-shaped cells (arrow) (H&E) (4×). C: Sheets of atypical spindle cells with elongated moderately pleomorphic hyperchromatic nuclei (arrows) (H&E) (40×). D: Sheets of malignant cells showing scattered mitotic figures (arrow) (H&E) (40×).

Subsequently, a radical cystoprostatectomy with ileal conduit was done. Intraoperatively, the urinary bladder was completely replaced by a hard mass which was excised, and subcentimeter lymph nodes were present along the right and left iliac vessels. Bilateral ureter, internal, and external iliac vessels were identified. Right and left pelvic lymphadenectomy was done. Bilateral superior and inferior vesical artery was identified and ligated. The urinary bladder is separated from the rectum, and the entire urinary bladder and the prostate and seminal vesicles are mobilized. The pubovesical plexus was ligated. The specimen was retrieved, and a fresh Foley catheter was placed as a drain. About 15 cm of the ileum has been divided and mobilized just 15 cm proximal to the ileocecal junction. End-to-end ileoileal anastomosis was done in four-layered techniques. Ureter cut ends spatulated and anastomosed to the ileum in separate ways with 4-0 Vicryl after bringing out 5-French-size infant feeding tubes as a stent. The distal end of the segment was brought out as stroma through the right iliac fossa. The proximal end was closed in two layers. Homeostasis was achieved, the drain was placed, and the wound was closed in layers. The specimen was sent for histopathological examination. Grossly, a radical cystoprostatectomy specimen measured 14×11×10 cm. The prostate measured 3×3×2 cm. The left and right ureters measured 7 cm and 2 cm in length, respectively. A tumor measuring 12×11×8 cm filling the lumen of the bladder arising from the right lateral wall of the bladder was seen (Figure 2A). The tumor was 4 cm and 10 cm from the right and left ureteric margins, respectively. Microscopically, sections showed a bladder wall lined by the urothelium and adjacent malignant tumor composed of sheets of oval- to spindle-shaped cells with moderate eosinophilic cytoplasm and moderately pleomorphic vesicular nuclei with prominent nucleoli, and few areas show increased mitoses (Figure 2B, 2C, 2D).

**Figure 3:**
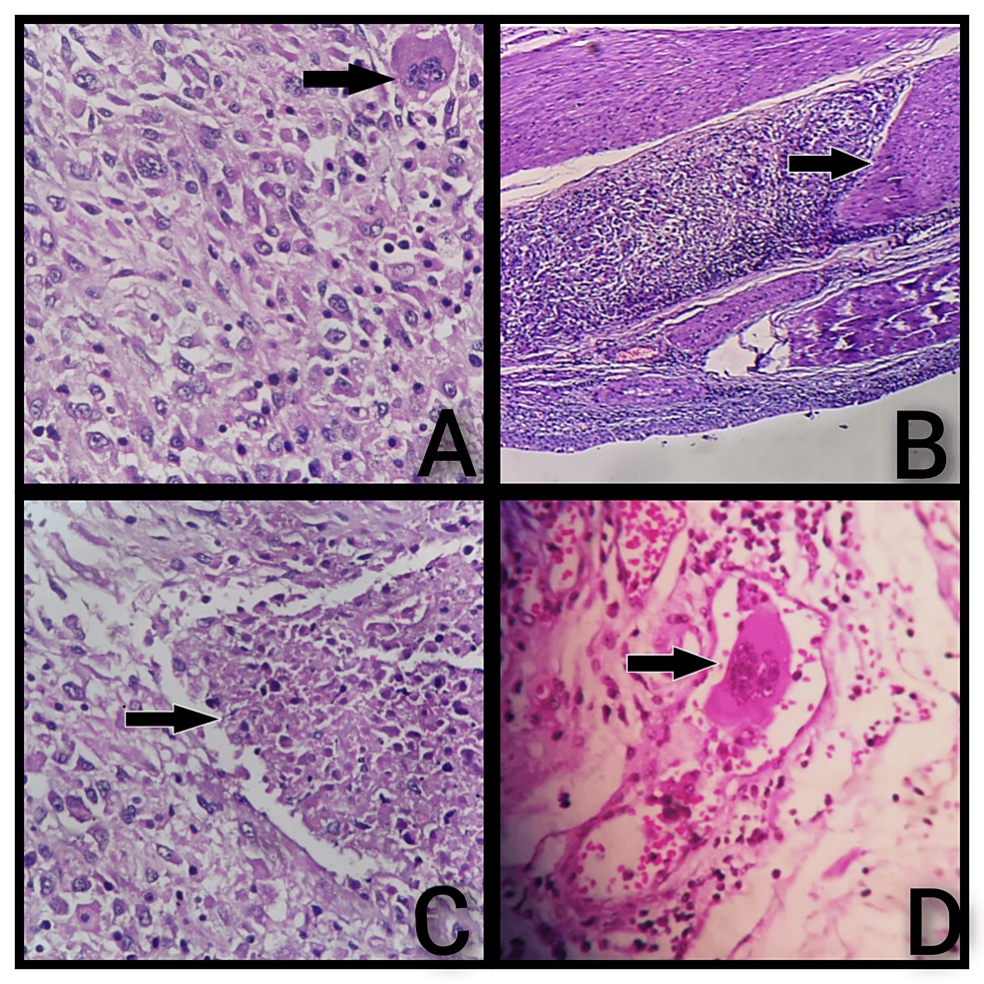
Microscopy of a sarcomatoid variant of urothelial carcinoma showing tumor giant cells, muscularis propria invasion, necrosis, and lymphovascular invasion. A: Multinucleated tumor giant cells (arrow) scattered amidst the tumor cells (H&E) (40×). B: Tumor infiltrating the superficial half of the muscularis propria (arrow) (H&E) (20×). C: Sheets of tumor cells with focal necrotic areas (arrow) (H&E) (40×). D: Lymphovascular invasion (arrow) (H&E) (40×).

Many areas show multinucleated tumor giant cells scattered amidst the tumor cells (Figure 3A). Areas of necrosis and focal lymphovascular invasion were also present (Figure 3C, 3D). The tumor cells are seen to invade the superficial half of the muscularis propria (Figure 3B). All margins are negative for tumor infiltration. All 12 resected bilateral pelvic lymph nodes were negative for metastatic deposits. According to the American Joint Committee on Cancer (AJCC) 8th edition, the tumor was classified as pT2a (superficial muscularis propria invasion was present). The N category was N0, as no nodal metastasis was identified. Thus, a final diagnosis of a high-grade SVUC of the bladder, pT2a pN0 was given. The postoperative period was uneventful. The patient was discharged with the following medications: Tab. Eltroxin, Tab. co-trimoxazole, and Ensure protein. The patient was doing well, and he intended to begin chemoradiotherapy after discussing it with a medical oncologist.

## Discussion

SVUC is an extremely rare variant, which accounts for only 0.1-0.3% of all urothelial carcinomas of the bladder. SVUC is distinguished by the presence of biphasic components; there can be morphological and/or immunohistochemical substantiation of epithelial and mesenchymal differentiation [4]. The patients with this variant have been associated with very poor disease-specific and overall survival rates in comparison with the high-grade pure urothelial carcinoma. According to the published literature, about 70% of the patients diagnosed with SVUC die within two years of diagnosis [5]. Patients with SVUC present with dysuria, nocturia, acute retention of urine, hematuria, and lower abdominal pain [2]. Grossly, these tumors usually appear bulky and polypoid. Microscopically, neoplastic cells show a variety of patterns. They are arranged in fascicles which gives a resemblance to leiomyosarcoma. Occasionally, there is a storiform pattern, evocative of unclassified pleomorphic sarcoma (malignant fibrous histiocytoma). Pleomorphic, elongated cells with moderate to abundant eosinophilic cytoplasm are seen, which is reminiscent of that seen in rhabdomyosarcoma [6]. SVUC usually presents with closely packed spindle cells that are separated by collagen fibers or loose myxoid stroma, with moderate to severe nuclear atypia. Increased mitotic figures are frequently seen. Sarcomatoid carcinoma frequently presents in the elderly; the ureter and renal pelvis may get involved sequentially. Metastasis can involve the regional nodes and distant organs [6]. The sarcomatoid areas are usually integrated with urothelial carcinoma, adenocarcinoma, and squamous cell carcinoma, resembling a high-grade sarcoma, which has not been otherwise specified [2]. The presence of heterologous differentiation such as osseous differentiation is seen in one-half of the cases, chondrosarcomatous differentiation is seen in one-third of the cases, and skeletal muscle differentiation (rhabdomyosarcoma) is seen in one-quarter of the cases. The differential diagnosis includes inflammatory myofibroblastic tumors, true sarcomas, and urothelial carcinomas with reactive stroma [2]. Liposarcomatous differentiation was reported in one case of SVUC. However, no definitive prognostic significance was found [2]. 

## Conclusions

Being a rare entity, it usually presents at a higher grade and is related to a dismal prognosis in comparison with conventional urothelial carcinoma. The prognosis of this tumor may be improved by early identification, radical surgery, and chemoradiotherapy. Tumors are resistant to conservative therapy, and carcinomas exhibiting muscularis propria invasion, regardless of grade, are managed with neoadjuvant chemotherapy, which is followed by a radical cystectomy and pelvic lymphadenectomy. However, to establish a definitive treatment approach, a multicenter trial is needed to investigate diverse treatment options for SVUC, to achieve consensus on the most optimal treatment strategy.
